# Can Multi-Label Classifiers Help Identify Subjectivity? A Deep Learning Approach to Classifying Cognitive Presence in MOOCs

**DOI:** 10.1007/s40593-022-00310-5

**Published:** 2022-09-02

**Authors:** Yuanyuan Hu, Claire Donald, Nasser Giacaman

**Affiliations:** Faculty of Engineering, The University of Auckland, Auckland, New Zealand

**Keywords:** Cognitive presence, Automatic text analysis, Multi-label classification, Online discussion, MOOC, BERT

## Abstract

This paper investigates using multi-label deep learning approach to extending the understanding of cognitive presence in MOOC discussions. Previous studies demonstrate the challenges of subjectivity in manual categorisation methods. Training automatic single-label classifiers may preserve this subjectivity. Using a triangulation approach, we developed a multi-label, fine-tuning BERT classifier to analyse cognitive presence to enrich results with state-of-the-art, single-label classifiers. We trained the multi-label classifiers on the MOOC discussion messages that were categorised into the same phase of cognitive presence by the expert coders, and tested the best-performing classifiers on the messages that the coders categorised into different phases. The results suggest that multi-label classifiers slightly outperformed the single-label classifiers, and the multi-label classifiers predicted the discussion messages as either one category or two adjacent categories of cognitive presence. No messages were tagged as non-adjacent categories by the multi-label classifier. This is an improvement compared to manual categorisation by our expert coders, who obtained non-adjacent categories and even three categories of cognitive presence in one message. In addition to the fully correct prediction, parts of messages were partially correctly predicted by the multi-label classifier. We report an in-depth quantitative and qualitative analysis of these messages in the paper. The automatic categorisation results suggest that the multi-label classifiers have the potential to help educators and researchers identify research subjectivity and tolerate the multiplicity in cognitive presence categorisation. This study contributes to extending the literature on understanding cognitive presence in MOOC discussions.

## Introduction

This paper explores using the multi-label deep learning approach to automatically analyse the phases of cognitive presence, as a triangulation approach to enriching the understanding of cognitive presence in the discussion messages in Massive Open Online Courses (MOOCs). As a type of open education, MOOCs have an absence of barriers to entry and a wider range of learner profiles, diverse learning objectives, and motivations. Tracing the first MOOC from 2008 (Siemens, 2013), MOOCs are booming in recent years (Lohr, 2020). The COVID-19 pandemic brings dramatic growth of learners and traffic to MOOCs (Shah, 2020a). In 2020, the new learners reached over 60 million in the leading MOOC platforms (e.g., Coursera, edX, Udemy combined), and over 2800 courses were launched by around 950 universities (Shah, 2020b). Nevertheless, these massive learners with various learning objectives lack direct instructions from the limited numbers of teachers in MOOCs (Kovanović et al., 2018). Effective and efficient feedback from instructors can encourage more learners participation and engagement, guiding learners to achieve their learning goals (Phan et al., 2016). MOOC educators also need support to monitor students’ learning progress and moderate instructional designs for engaging the students and promoting their deep and meaningful learning (Yousef et al., 2015). Facilitating the educator-learners’ dialogue is an ongoing challenge for MOOC designers, educators and stakeholders. MOOC educators struggle to provide personalised feedback to the large number of learners enrolled, with unique learning goals, without the support of computer algorithms.

Asynchronous online discussion forums are a vital component of MOOCs, offering a virtual place for learners and instructors to interact and communicate together. Previous studies found that learners who actively engaged in discussion forums tend to achieve better performance in MOOCs (Tang et al., 2018; Wise & Cui, 2018). Analysing the discussion messages in MOOCs can contribute to understanding learners' critical discourse (i.e., critical thinking, higher-order thinking, and cognitive presence), which is beneficial for instructors and course designers to adjust the course content and teaching strategies accordingly. Scholars have proposed theoretical frameworks and measurement tools (Gunawardena et al., 1997; Newman et al., 1995) to investigate critical discourse in online discussion messages. Among these, the Community of Inquiry (CoI) framework proposed by Garrison et al. (1999) was the most widely used framework to analyse learning in online courses for over two decades. As a core dimension of the CoI, cognitive presence reflects learners' critical discourse, which is closely correlated to the progress of knowledge (re)construction during learning. The classification rubric of cognitive presence (Garrison et al., 2001) was also adapted for exploring the depths of critical discourse in different educational contexts (Hu et al., 2020; Park, 2009).

The manual classification of cognitive presence can be practical for the limited discussion messages in small-scale online courses, but not feasible to analyse the myriad messages in MOOCs. Previous studies suggested the coders had difficulty distinguishing adjacent phases of cognitive presence in MOOC discussions (Hu et al., 2020, 2021a; Kaul et al., 2018). The main causes of the disagreements in the manual categorisation process could be the ambiguities of conversational language used and the vastly increasing data scale in the MOOC discussion threads (Hu et al., 2021a). It tends to be subjective in the process of interpreting other people’s comments into different categories of cognitive presence by a person (Park, 2009), since the natural languages (human languages) have ambiguities (Jackson, 2020). More coders’ participation can reduce the subjectivity through training and negotiation. However, it is impossible for us to employ unlimited coders for the manual categorisation work. Automatic classifiers can be the assistants to the human coders to analyse the cognitive presence in MOOC discussions.

Some automated methods for analysing cognitive presence have also been developed to discover the indicators of the progressive cognitive phases in online discussion messages (Barbosa et al., 2020; Corich et al., 2006; Farrow et al., 2020; Kovanović et al., 2016; McKlin et al., 2001; Neto et al., 2018). These automated methods built single-label classifiers, which had the risk to inherit the subjectivity from the manual categorisation work in the training data preparation stage. The traditional machine learning (e.g., random forest) used in the previous studies can only classify an instance (e.g., a discussion message) into an exclusive class in the multi-class categorisation problems. To prepare more training data, the coders addressed the ‘problematic’ messages subjectively after negotiations to reach a 100% agreement in the previous studies (Kovanović et al., 2016; Neto et al., 2018). The ‘problematic’ messages were also trained to have one single category: therefore, the subjectivity of the manual categorisation may have been preserved in the automatic single-label classifiers to some extent. Another problem is that the previous studies were in the context of the traditional, small-scale, for-credit university courses. The discussion messages in the courses can be easily classified into one single phase of cognitive presence as the coding rubric of the CoI framework was developed from the messages in the similar context (Garrison et al., 2001). However, the messiness, informality and disordered in the vast large-scale discussion messages in MOOCs are far more complex than in the coherent, more structured messages in the small-scale discussions (Almatrafi et al., 2018; Hu et al., 2020). The taxonomies with clear boundaries between the cognitive presence categories may not be applicable for analysing the MOOC discussions (Hu et al., 2020). Using a triangulation approach, we applied multi-label classification methods to analyse cognitive presence in MOOC discussions.

The multi-label classification (prediction) can allow a text associated with more than one label simultaneously, which has been used in the semantic scene classification (Shen et al., 2004), multimedia classification (Trohidis et al., 2011), and sentiment analysis tasks (Liu & Chen, 2015; Tang et al., 2020). By using the multi-label classification methods, discussion messages can be labelled more than one category with probabilities rather than enforcing them into a consensual label. In this study, we trained multi-label classifiers with the MOOC discussion messages that have been manually labelled as the same cognitive category by the expert coders without confusion. We are interested in the performance of the multi-label classifiers to predict the discussion messages with one or multiple labels in the test set, shining a light on the refinement of the CoI frameworks in MOOCs. Thus, the *main research question* in this study was: *What would the prediction performance of using the multi-label classifiers to categorise the phases of cognitive presence, and what can we learn about cognitive presence in the MOOC discussion messages from the training and test processes?* To answer this main question, we proposed three sub-questions as below:

- *Sub-question 1 (SQ1):* To what extent can our multi-label classifier accurately predict the cognitive presence categories in online discussion messages from a target MOOC? Can the multi-label deep learning classifier outperform the state-of-the-art, single-label classifier in this study?
- *Sub-question 2 (SQ2):* Are there any MOOC discussion messages, which have been manually labelled into the same category by coders, be tagged into multiple labels by the multi-label classifier? In what proportion? Are there any common patterns?
- *Sub-question3* (SQ3): Are there any MOOC discussion messages, which have been manually labelled into different categories by coders, be tagged into one single label by the multi-label classifier? In what proportion? Are there any common patterns?

## Background

### The Community of Inquiry Framework and Cognitive Presence

In the past two decades, the Community of Inquiry framework proposed by Garrison et al. (1999) has been most broadly used to analyse learning in asynchronous online discussion forums. The CoI portrays the educational experience that occurs in a virtual learning community, in which ‘*a group of individuals who collaboratively engage in purposeful critical discourse and reflection to construct personal meaning and confirm mutual understanding*’ (Garrison & Anderson, 2011, p.2). The CoI framework defines three interdependent dimensions, also called presences, to analyse learning engagement: 1) *Cognitive presence*, as a primary dimension of the CoI, depicts the critical discourse and reflection of knowledge (re)construction and problem-solving processes (Garrison et al., 2001); 2) *Social presence* represents social climate and interpersonal communications between the participants of online discussions (Rourke et al., 1999); 3) *Teaching presence* analyses the instructional activities that direct and moderate the discussions (Anderson et al., 2001).

**Fig. 1:**
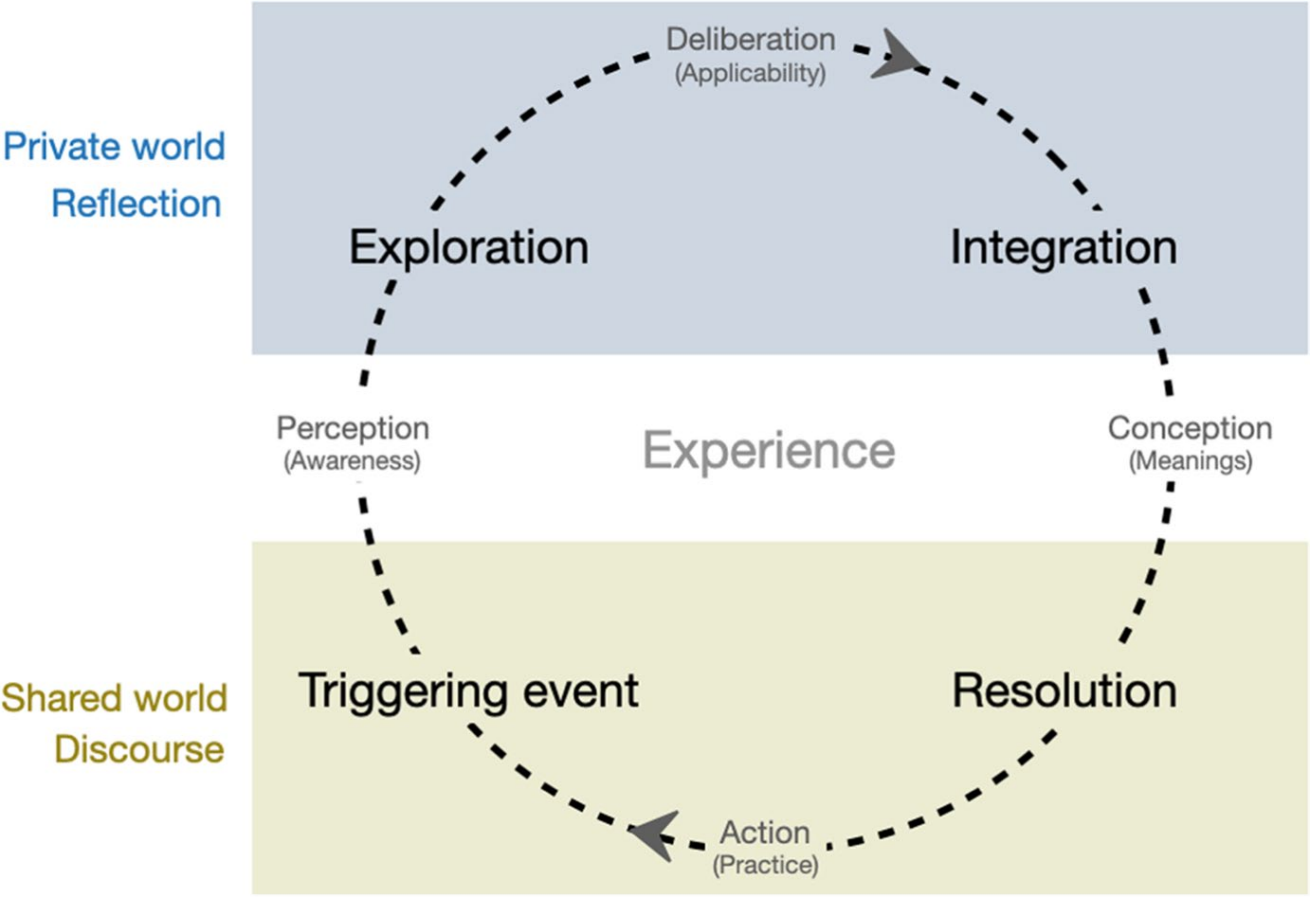
The Practical Inquiry Model (PIM) demonstrating the Four Phases of Cognitive Presence in a Learning Community. The diagram was adapted from Garrison et al. (2001)

This study concentrates on analysing the construction and facilitation of learners' cognitive presence in MOOC discussions. The cognitive presence is the paramount evidence that students actually learn about the knowledge concepts in the domain, so it needs to be investigated before the other dimensions (Rourke & Kanuka, 2009). As shown in Fig. 1, four phases of cognitive presence are defined in a cycle of progressive knowledge construction (Garrison et al., 2001): 1) *Triggering event*, in which the learners propose their questions or confusions usually as a trigger of a thread; 2) *Exploration*, in which the learners exchange information to investigate the answers or solutions of the problems proposed in the previous phases but not able to reach a coherent conclusion; 3) *Integration*, in which the learners synthesise coherent conclusions to the problems proposed in the previous phases with sufficient support; 4) *Resolution*, in which the learners apply, test or argue the solutions or conclusions suggested in the previous phase, shaping the new meaning or construct. We acknowledge the richness and complexity of message content in the MOOC discussion data. Since this study focuses on the cognitive presence, messages that were not fitted into any of the above phases were classified as the *Other* category. For example, messages that only indicate social presence (i.e., ﻿emotional communication to enrich interpersonal relationships in the online community) were classified into *Other*. The other two presences of the CoI (social and teaching presences) will be explored in our future work. We applied an adapted coding rubric of cognitive presence (Hu et al., 2020) to classify the discussion messages in the target MOOC. Following the *coding-up* rule recommended by Garrison et al. (2001), a message that reflected the indicators of more than one cognitive category was classified into the highest one. Table 1 provides examples of the messages that were classified into the five categories of cognitive presence in a thread.

**Table 1:** Message instances of the five categories in cognitive presence in a thread

	The message text	Category	Brief definition
The post	How could I apply the (non)deductive concept in the Cognitive Psychology?	Triggering event	Messages that present learners’ confusion
The 1^st^ reply	After the a given experiment, we normally find some evidence that are not exactly true, but it could occur whether these are tested. In this instance, I should direct my arguments into non-deductive perspective	Exploration	Messages that exchange information to investigate solutions to the questions but without a coherent conclusion
The 2^nd^ reply	I would have thought that this situation would be a statistical analysis—say a Chi-square against nominal potential outcomes, for example, True, False and Unknown against a frequency of outcome. Any statistical analysis is essentially non-deductive since the output is always based upon probabilities. The confidence level, say 95%, is the threshold one would use to determine plausibility based upon an evaluated p Value	Integration	Messages that provide coherent solutions to improve the problem with sufficient substantiation
The 3^rd^ reply	Even then, there will always be assumptions about the underlying system involved in using a test statistic. It is the experimental design which should govern what outcomes can be expected whilst taking into account which analysis methodology should be used and, therefore, what assumptions underlie the hypotheses but, regardless, if one relies upon statistical analysis of a sample, the argument is non-deductive—an induction based upon the probability of refuting a hypothesis	Resolution	Messages that apply, test or, argue the previous solutions to construct new ideas
The 4^th^ reply	Thank you so much for the comments	Other	Messages that do not fit into any of the above categories

### Machine Learning Classifiers of Cognitive Presence in Online Discussion Messages

Several studies have developed automated classifiers using different algorithms to analyse cognitive presence in online discussion messages. However, the majority of the discussion messages were from the traditional, small-scale, for-credit online courses rather than MOOCs, and they were tagged into a unique category by the automatic classifiers.

Initially, McKlin (2004) and Corich et al. (2006) developed a simple artificial neural network and a Bayesian network to classify cognitive presence, respectively, using dictionary-based words as classification features. McKlin’s (2004) classifier reached the Holsti’s coefficient of reliability (CR) of 0.68 and Cohen's κ of 0.31, whereas Corich et al.’s (2006) classifier achieved the CR of 0.71 without any report of Cohen’s κ. To improve the classification performance, Kovanović et al. (2014) developed a Support-Vector-Machine (SVM) classifier using n-gram and structural features as classification indicators. The SVM classifier performed the best accuracy of 58.4% and Cohen’s κ of 0.41. To emphasise the importance of the structural features for identifying cognitive presence phases, Waters et al. (2015) built a Conditional Random Field classifier based on Kovanović et al.’s (2014) methods. The best accuracy and Cohen’s κ were increased to 64.2% and 0.482.

However, using the n-grams or dictionary-based words as classification features revealed two weaknesses: 1) a large number of features constructed a high-dimensional space that could easily raise over-fitting problems; 2) the resulting classifiers were domain-specific; thus, the classifiers were difficult to generalise to other domains. To address these issues, Kovanović et al. (2016) developed a Random Forest (RF) classifier by using the features mainly extracted from two computational linguistics tools, Coh-Metrix (McNamara & Graesser, 2013) and Linguistic Inquiry Word Count (LIWC, Tausczik & Pennebaker, 2010). This RF classifier also applied over-sampling methods to improve the class imbalance problem caused by the skewed distribution of cognitive presence categories in the sample data. The RF classifier performed the best accuracy of 70.3% and Cohen’s κ of 0.63, which is state of the art. Nonetheless, a replication study by Farrow et al. (2019) pinpoints that Kovanović et al.’ (2016) method obtained over-optimistic results as it applied the over-sampling method before the training-test data split. After Farrow et al. (2019) employed the over-sampling method merely for the training process; the best accuracy decreased to 61.7% and Cohen’s κ to 0.46. The random forest approach was also applied to build automated classifiers for analysing cognitive presence in cross-language discussion messages. Neto et al.’s (2018) study reached the accuracy of 83% and Cohen’s κ of 0.72 in the Portuguese discussion data, and Barbosa et al.’ (2020) study achieved the accuracy of 67% and Cohen’s κ of 0.32 in cross-language (English & Portuguese) discussion data. Barbosa et al. (2021) then extended the RF classifier using automatic text translation method to analyse both cognitive and social presence in the cross-language discussion messages and obtained similar performance results to the previous studies. Neto et al. (2021) also applied the RF classifiers to classify the discussion messages from two discipline courses (biology & technology), reaching Cohen’s κ of 0.55 in the experiments of using combined data sets and the Cohen’s κ of below 0.4 in the cross-discipline tests.

### Deep Learning and Multi-Label Classifiers for MOOC Discussions

Deep learning algorithms have been shown outstanding performance in many fields, especially in computer vision fields (Voulodimos et al., 2018). Its applications have also exploded in the Natural Language Processing (NLP) field over the last years (Otter et al., 2021). Deep learning algorithms investigate intricate patterns in large data sets using the backpropagation methods to optimise the internal parameters of the neural networks that aim for high and robust performance (LeCun et al., 2015). A deep learning approach was adopted to categorise phases of cognitive presence in online discussions including both for-credit and MOOC data, reaching the results up to Cohen’s κ of 0.528, a similar performance to the application studies of the RF classifiers (Hu et al., 2021b).

As a type of deep Recurrent Neural Network (RNN), the Transformer aims to solve the problems where the sequential input information is required to pass to the output sequences, such as speech recognition and machine translation tasks (Vaswani et al., 2017). It fulfils the drawbacks of the traditional RNNs that are prone to forgetting or mixing the content of distant-position information in sequence. It is gaining popularity in NLP fields. Bidirectional Encoder Representation from Transformers (BERT), as a language model built on the Transformer, has dramatically improved the state of the art in a wide range of NLP tasks (Devlin et al., 2019; Liu et al., 2019). Google has provided the BERT models pre-trained by the large corpora (Wikipedia and Book Corpus) for developers and researchers to fine-tune and apply in their personalised tasks. In learning analytics fields, the BERT model shows great potential to analyse teacher discourse in online classrooms automatically (Jensen et al., 2021). The fine-tuning BERT model was applied to analyse cognitive presence in discussion messages from a computer science MOOC and for-credit course, which reported the promising *F*_*1*_ scores of 0.95 (Hosmer & Lee, 2021; Lee et al., 2022).

The BERT model can also be applied to construct multi-label classifiers for text classification tasks. A fine-tuned BERT model for multi-label tweets classification demonstrates promising performance (Zahera et al., 2019). In learning analytics fields, a fine-tuned BERT model for multi-label sentiment analysis in multilingual texts also reveals higher performance than the previous machine learning models (Tang et al., 2020). We thus wonder, how well can the fine-tuned BERT model perform on the multi-label classification of cognitive presence in the target MOOC discussion data.

## Methods

### Data Description

The data set used in this study came from an archived offering of the Logical and Critical Thinking (LCT) MOOC on the FutureLearn platform.^1^ This MOOC was taught and designed by a team from a New Zealand university. The course was an introductory Philosophy MOOC, which focused on the basic concepts of effective thinking, how to build valid arguments, and how to link the arguments with daily life. Each course offering served eight weekly topics with several learning tasks in different formats, such as lecture videos, articles, quizzes, and discussion boards. Registered learners can leave their comments under all the learning tasks, excluding quizzes. Each course offering had approximately 11,000 registered learners and 12,000 discussion messages (e.g., an individual message refers to a starting post or its replies in each discussion thread). To reduce the impacts from different content of learning tasks, we randomly selected 16 tasks (two from each week). Then, a sample of around 100 messages was randomly selected from each of the 16 tasks. An entire thread with all its sequential replies was maintained in the selection process rather than detached to achieve an exact number of samples. For example, we selected 103 messages randomly from the third task, and the 103 messages consisted of 41 threads that contained 41 posts and all the replies underneath, such as the post and its four replies displayed in Table 1. In this way, our sample data were composed of 1,917 discussion messages from the learners in the target MOOC.

Three expert coders classified the sample data into four phases of cognitive presence and the Other (the fifth category), according to an adapted coding rubric for the MOOC discussions (Hu et al., 2020). The coders were trained round by round (50 new messages per round) before independently achieving an over 80% agreement without negotiations. A percentage agreement of 81% was reached between them in the third round, and then they were allocated the 1,917 messages to classify. Finally, they reached an overall percentage agreement of 77.15% and Fleiss’ κ of 0.763. Table 2 displays the distribution of the five cognitive presence categories in the sample data. This distribution is consistent with the findings in most of the previous studies on the classification of cognitive presence in online discussions (Hu et al., 2020, 2021a; Kaul et al., 2018; Kovanović et al., 2014; Park, 2009; Rourke & Kanuka, 2009), where the bulk of messages were in Exploration, and small fractions in Resolution and the Other.

**Table 2:** Agreements between the three coders by cognitive presence phases in the sample data (1,479 discussion messages as the AgreementSet)

Cognitive phase	Messages	
	*n*	%
Other	85	5.75
Triggering event	279	18.86
Exploration	835	56.46
Integration	244	16.50
Resolution	36	2.43

**Fig. 2:**
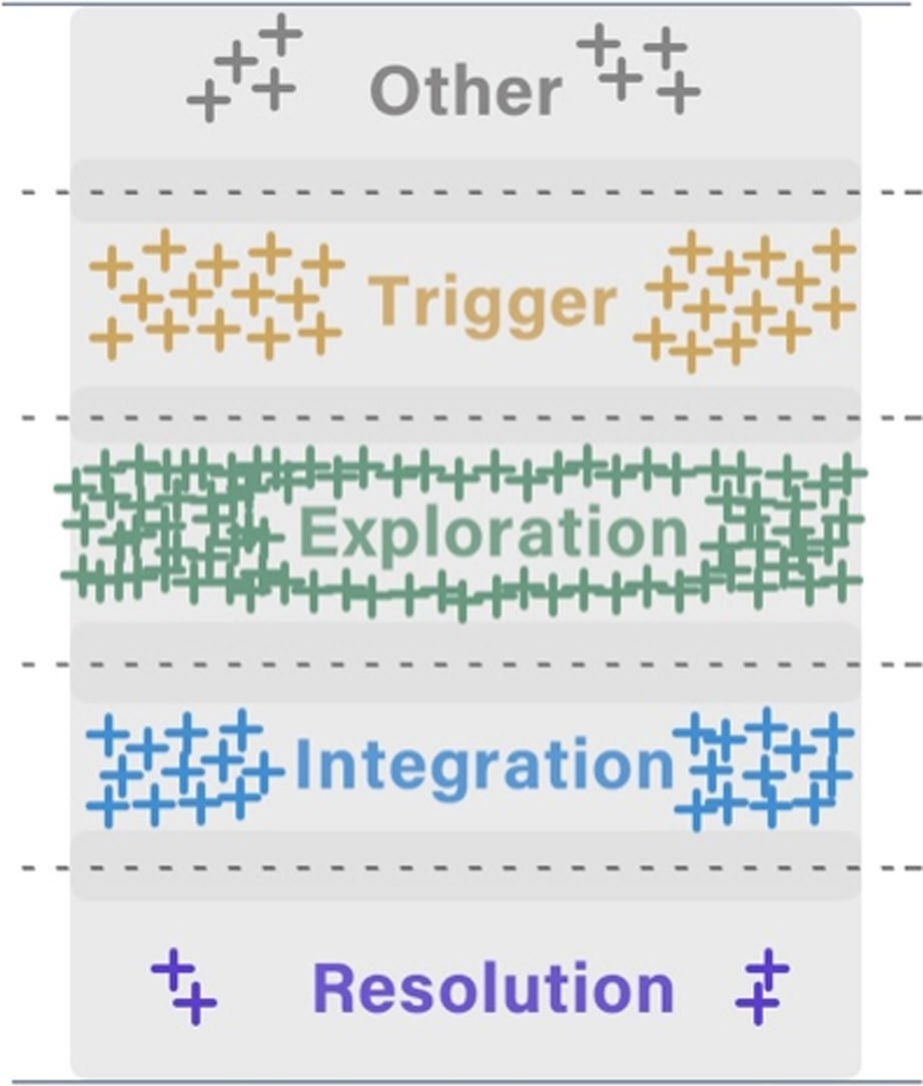
A conceptual diagram representing the relative positions of the sample messages in the five cognitive presence categories

In this study, we regard the manually classified messages as ordinal data, as it obtains the ordering of classes from the lowest to highest (Hildebrand et al., 1977, p.5). A conceptual diagram (Fig. 2) aligned to Table 2, depicts the relation of the messages within the five cognitive presence categories (i.e., their placement vertically depicts their strength relative to the respective categories). As only the agreement messages were used, the markers were seated within the exact area of each category, without any overlaps with adjacent categories. To simplify the number of markers in Fig. 2, each plus marker represents approximately ten messages. Five colours differentiate the cognitive presence categories in Fig. 2. We call this part of data AgreementSet for short in the rest of the article.

**Fig. 3:**
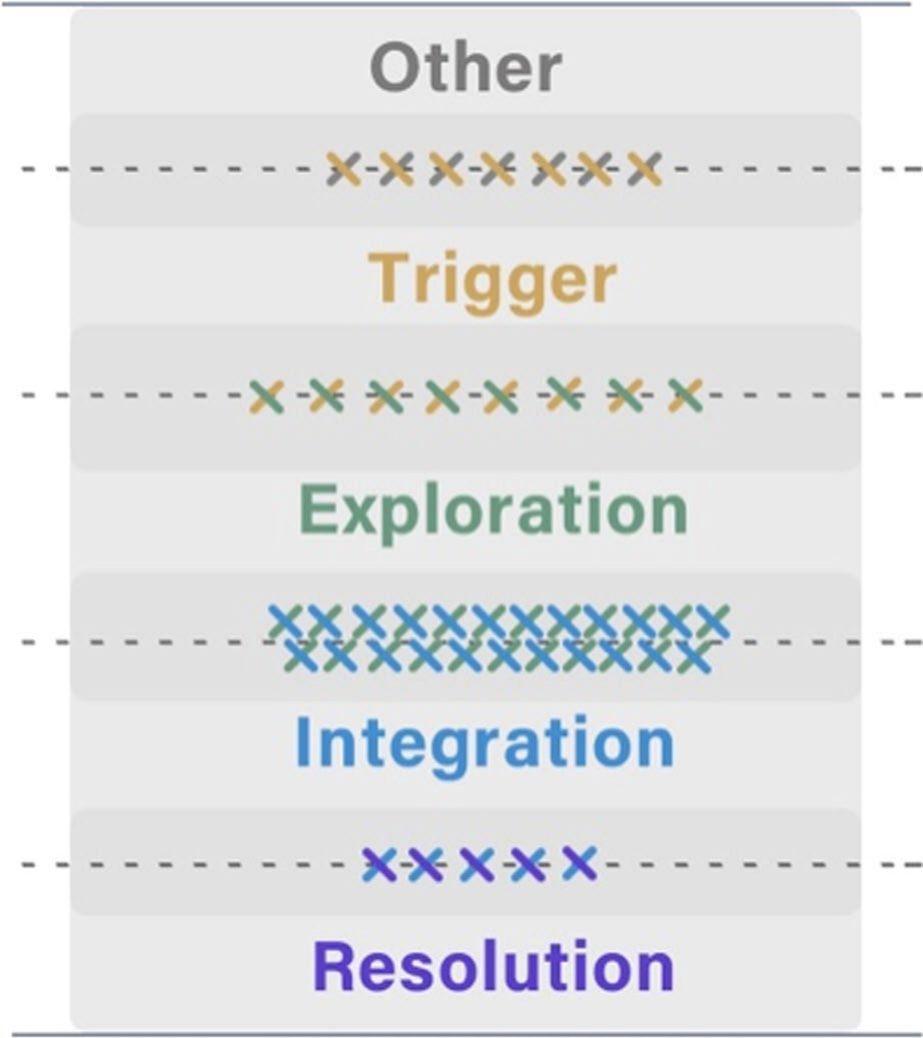
A conceptual diagram representing how the messages about which coders disagreed were situated between adjacent categories of cognitive presence

Table 3 shows the distribution of cognitive presence categories in the coders' disagreements (i.e., messages that were manually labelled differently). Most of the disagreements had two labels, with more than half labelled between Exploration and Integration by the coders. A conceptual diagram Fig. 3 aligned to Table 3 depicts how the disagreements were situated in the overlapping areas between adjacent categories of cognitive presence. Similarly, each cross marker represents around ten messages so that the groups with less than ten messages were not shown in Fig. 3. Each cross has two lines of different colours, representing the two labels of cognitive presence categories within the disagreements. We name this part of data DisagreementSet for short.

**Table 3:** Disagreements between the three coders by the cognitive presence phases (438 messages as the DisagreementSet)

Cognitive phases	Messages	
	*n*	%
Other & Trigger	66	15.07
Trigger & Exploration	78	17.81
Exploration & Integration	227	51.83
Integration & Resolution	50	11.42
Exploration & Resolution	4	0.91
Other & Exploration	7	1.60
Three phases	6	1.37

In this study, the multi-label classifiers were first trained and tested on the AgreementSet (Table 2) to answer our *SQ1* and *SQ2*. We used the majority of the AgreementSet data as the training data because the coders could easily classify them without confusion. The DisagreementSet data, in which the coders disagreed about which categories the messages belonged to, would confuse the computer algorithm more if we included them in the training process. The DisagreementSet data were used to validate the best-performing multi-label classifiers to answer the *SQ3* and to enrich our understanding of cognitive presence in MOOC discussions.

### Multi-Label Classifier Architecture and Procedures

The multi-label classification in this study denotes that more than one category can be tagged to a discussion message. The output of the multi-label classifier is an array of five scores between 0 and 1, which indicates the probability of a message being each cognitive presence category. A common solution for the multi-label classification tasks is the problem transformation method (Alazaidah et al., 2015). It transforms the multi-label classification problem into the single-label classification problem, which can employ single-label classifiers. Thus, we revised the standard BERT model from a class-independent model to five binary BERT classifiers for each category. A similar architecture was employed in a previous study on multi-label sentiment analysis in online monolingual discussion texts, which presented a higher prediction performance than the machine learning models (Tang et al., 2020).

**Fig. 4:**
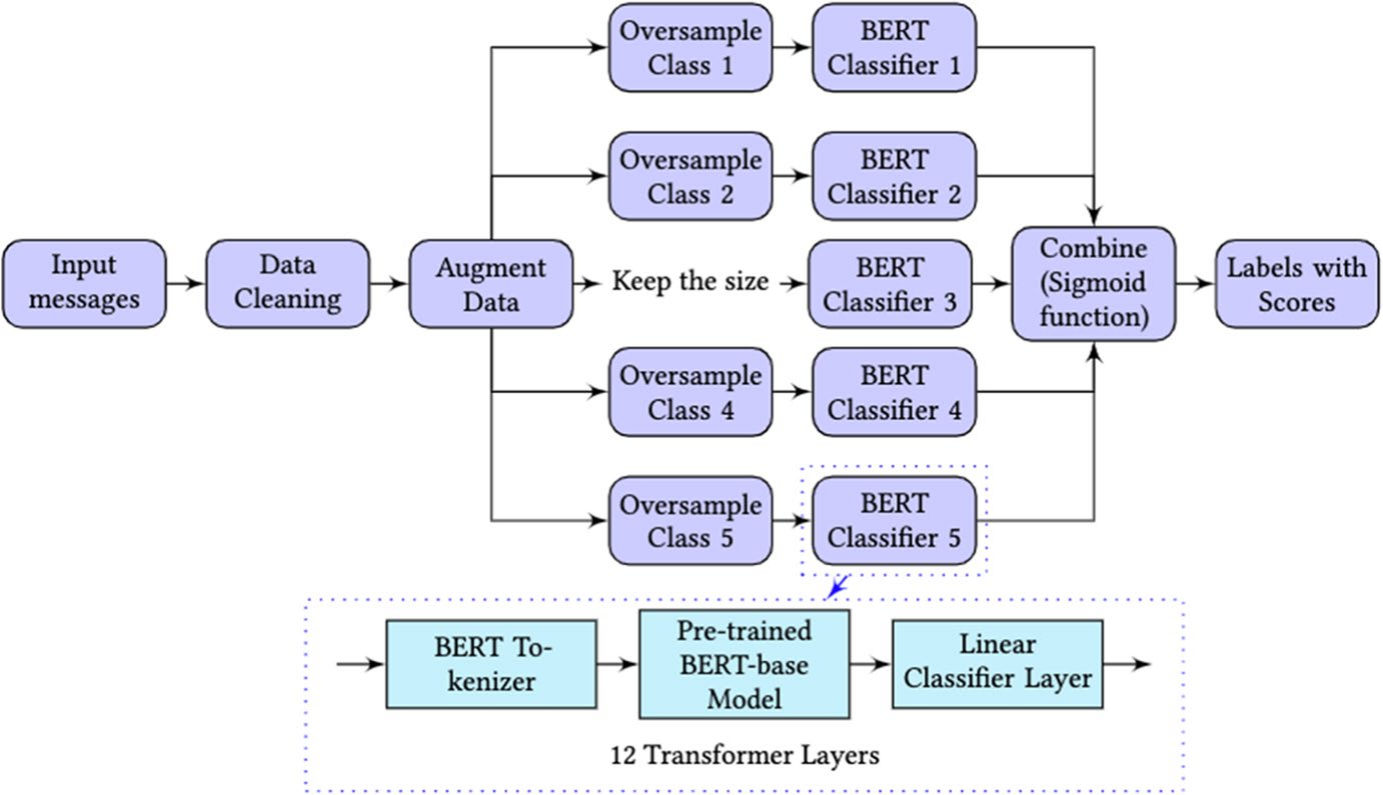
The architecture of the multi-label classifier for analysing the cognitive presence phases

Figure 4 displays the architecture of the multi-label, fine-tuned BERT model in this study. In the pre-processing stage, we replaced all the emojis or URLs into a word ‘EMOJI’ or ‘URL’, expanded abbreviations (e.g., I’d to I would), and removed repeated characters, redundant spaces and stop words for every message. After data cleaning, the tenfold Cross-Validation (CV) method was applied to randomly divide the entire sample data into ten non-overlapping folds of approximately equal size using the stratified sampling method. The nine-fold data was the training set in every CV loop, and the remainder was the testing set. We then repeated the entire training and testing process ten times to reduce the risk of overfittings. In each training loop, we split the nine-fold data into training and validation partitions in a 9:1 ratio to fine-tune the best model. We also found that the classes of the sample data were unbalanced, which may impact the classification performance. To address this problem, we applied the data augmentation method to over-sample the smaller classes in the training set before feeding them into the multi-label classifier. The data augmentation and the over-sampling procedures proceeded after the initial dataset splits in each training loop to avoid over-optimistic results caused by the data contamination risk as recommended in Farrow et al. (2019). More details of the data augmentation and the over-sampling method are provided in Data augmentation and over-sampling method Section.

Figure 4 also shows the structure of the binary BERT classifier for each cognitive presence category. After the augmentation process, the messages were converted into tokens by BertTokenizer. Then, the tokens of messages were fitted into a pre-trained BERT model. We used the *bert-base-uncased* model constructed by 12 transformer layers with 768 dimensions (Devlin et al., 2019). We added a linear classifier layer that transferred the BERT representations into classification tasks after the BERT model. Finally, a sigmoid function was performed to output the predicted scores of five cognitive phases ranging from 0 to 1. The sigmoid layer can allow more than one phase to reach a score of over 0.5 so that multiple labels may appear at a message simultaneously. The parameters of our best-performing model were using a batch size of 8, epochs number of 10, and a learning rate of $2\times {10}^{-5}$. We used Binary Cross-Entropy to calculate the error rate of each label.

### Data Augmentation and Over-Sampling Method

The data augmentation method aims to increase the data size of the smaller categories for better model performance (Dyk & Meng, 2012). Compared to creating augmented images in computer vision fields, text augmentation in NLP tasks is much more difficult due to the complexity of human language (Fadaee et al., 2017). We adopted the *nlpaug* (Ma, 2019) approaches to generate synthetic data for the minority categories in the training data set. We applied multiple augmentation methods, including character-level and word-level augmenters from the *nlpaug* library, and combined them as sequential pipelines to generate a diversity of over-sampling data set. The character-level augmenters can add spelling errors into one or more words in a text; for example, changing ‘dog’ to ‘d0g’ or ‘eat’ to ‘eta’ in a sentence. The word-level augmenters can find semantically similar words by different word embeddings models (e.g., *Word2Vec* (Mikolov et al., 2013) and *GloVe* (Pennington et al., 2014)) to replace the original words in the text for generating new instances.

**Fig. 5:**
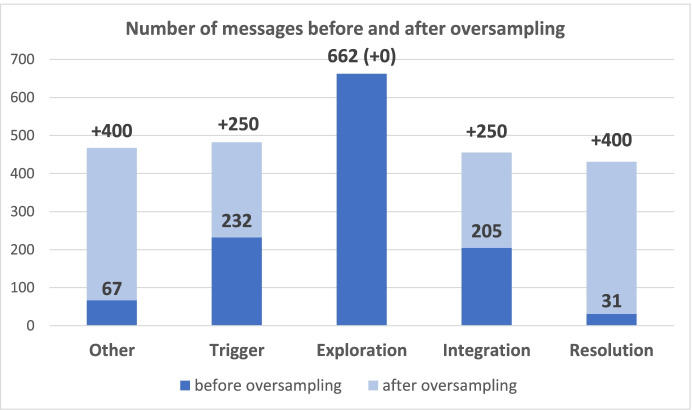
Number of messages before and after oversampling across five cognitive phases

We found increasing the minority categories by a certain ratio can achieve better prediction performance than reaching the same number of messages in the largest category (i.e., Exploration) by the different trials of oversampling ratios in the training process. In the best case, we created 400 synthetic messages for the fewest categories (i.e., the Other and Resolution), and 250 for the second-fewest categories (i.e., Triggering event and Integration), and maintained the original size of the largest category (i.e., Exploration) as shown in Fig. 5.

### Evaluation Metrics

Accuracy, precision, recall and *F*_*1*_ scores are common evaluation metrics for single-label classification tasks (Tharwat, 2020). However, the predicted labels of each instance are a set rather than a single item in multi-label predictions. The metrics extended from the single-label measures were used to validly distinguish the notions of *fully correct*, *partially correct* and *fully incorrect* (Sorower, 2010). In this study, we adopted the *exact match ratio (MR)* (Sorower, 2010) and *example-based F*_*1*_* score* (Herrera et al., 2016, p.56) as two common metrics used in the multi-label classification problems (Ceylan & Pekel, 2017; Du et al., 2019; Pereira et al., 2018). The *example-based* measures aim to capture the differences between actual labels (i.e., the manual labels) and the predicted labels for each test instance (i.e., the test message) separately, and then average over all test instances in the test set (Sorower, 2010). The *MR* focuses on *fully correct* prediction ratios regardless of the *partial correctness*, whereas the *example-based F*_*1*_* score* accounts for the *partial correctness* of the prediction sets (Godbole & Sarawagi, 2004). By comparison, the same calculations were used to measure the baseline single-label classifiers as the two metrics are extended from the accuracy score and *F*_*1*_* scores* in single-label classification problems (Sorower, 2010).

Apart from the *fully correct* predictions, the *partially correct* predictions are also worth taking into consideration. A test message is defined as a *partially correct* prediction if the number of the predicted labels is more than one and one of them is equal to the manual label in this study. According to the definition of the *MR* (Sorower, 2010), we defined the *partially correct ratio (PR)* as

$$PR =\frac{1}{n}{\sum }_{i=1}^{n}(I({Y}_{i} \cap \widehat{{Y}_{i }})-I({Y}_{i}==\widehat{{Y}_{i }}))$$

where a multi-label data set contains $n$ instances ($1\le i\le n$) and then each instance is ${x}_{i}$ (${x}_{i}\in \chi$), and the manual labels set for $i$ th instance is ${Y}_{i}$ $({Y}_{i}\in \mathcal{Y}={\{0, 1\}}^{k}$, $k$ is the number of labels), and a prediction-label set $\widehat{{Y}_{i}}(\widehat{{Y}_{i}}={\{0, 1\}}^{k})$. Similarly, a test message is a *fully incorrect* prediction if neither of the two predicted labels is equivalent to the manual label; thus, we defined the *fully incorrect ratio (IR)* as

$$IR =\frac{1}{n}{\sum }_{i=1}^{n}(I({Y}_{i}\ne \widehat{{Y}_{i }}))$$

The computation of *IR* is equivalent to the *error rate* in the single-label classifications as defined in Tharwat’s (2020) work.

We also evaluate the prediction performance of the classifiers on each individual category. For each cognitive presence category, we compared the *macro-averaged F*_*1*_* scores* (Tharwat, 2020) of the multi-label classifiers to the baseline single-label classifiers. Similar metrics were adopted in previous multi-label classification of sentiment analysis studies (Liu & Chen, 2015; Tang et al., 2020). We used the macro-averaged *F*_*1*_ rather than the *micro-averaged score* since we regarded every category as equally important.

### Random Forest Classifiers as a Baseline

We also performed a Random Forest (RF) approach as a baseline compared to the multi-label classifiers. Only the single-label classification approaches have been attempted in the literature of automatic classification of cognitive presence in online discussions. The RF approach and its extensive use was regarded as the state-of-the-art single-label classifier for analysing cognitive presence in online discussions (Barbosa et al., 2021; Farrow et al., 2019; Kovanović et al., 2016; Neto et al., 2018).

Followed the previous studies, we adopted 199 classification features extracted from the sample data by two computational linguistics tools, Coh-Metrix (McNamara & Graesser, 2013) and LIWC (Tausczik & Pennebaker, 2009). Some contextual features, such as message depth and the number of replies, were excluded for consistency with the multi-label classifiers. Also, we applied the SMOTE (Synthetic Minority Over-sampling Technique) exact method (Farrow et al., 2019) to address the class imbalance. The sample data was pre-processed with the same approach as described in Sect. 3.2. After the pre-processing and feature extraction steps, a tenfold CV method was used in the entire sample data to select an optimal RF model through fine-tuning the two vital parameters, *mtry* (i.e., the number of classification features chose by each decision tree) and *ntree* (i.e., the number of decision trees). After fine-tuning, *mtry* of 118 and *ntree* of 700 were reported as the optimal parameters in the baselines. A final tenfold CV method was performed to repeat the training-test loop ten times with the optimal RF classifier to maintain consistency with the multi-label classifiers. We compared the classifier performance with and without the SMOTE exact method to the multi-label classifiers with and without the data augmentation methods, respectively, in Sect. 4.1.

## Results and Discussion

### Evaluation of the Multi-Label Classifiers for Identifying Cognitive Presence—SQ1

We report the prediction performance of the multi-label classifiers compared to the single-label baselines in this section. The initial output of the multi-label classifiers for each message instance was a vector of prediction scores between 0 and 1 in the five categories. To compute the evaluation metrics, the prediction scores were converted into labels of 0 or 1 with a threshold. In this study, a threshold of 0.33 was selected since the weight that a human coder tagged a cognitive presence label over the three coders' decisions was equivalent to around 0.33.

### Training and Testing on the AgreementSet

Table 4 demonstrates the *example-based* metrics (the best case and the SDs) of the multi-label classifiers with and without the Data Augmentation (DA) method to compare with the single-label baselines (RF classifiers). The multi-label classifiers achieved the lower *fully incorrect* ratios but slightly higher *example-based F*_*1*_ scores than the single-label baselines. Instead of *fully correct* or *incorrect*, several messages fell into the *partially correct* group by the multi-label classifiers. Also, the use of the DA method enlarged the proportion of the *partially correct* group from 12.16% to 39.86%.

**Table 4:** The overall example-based performance of the classifiers (the best case and the SDs on the testing AgreementSet)

Classifiers	Exact match ratio % (SD)	Partially correct ratio % (SD)	Fully incorrect ratio % (SD)	Example-based F_1_ score (SD)
Multi-label BERT without DA	**68.24** (0.04)	12.16 (0.03)	19.59 (0.03)	**0.77** (0.04)
Multi-label BERT with DA	45.95 (0.03)	**39.86** (0.03)	**14.19** (0.02)	0.75 (0.04)
Single-label RF without SMOTE	**73.60 (0.03)**	-	26.35 (0.03)	0.73 (0.04)
Single-label RF with SMOTE	73.00 (0.05)	-	27.00 (0.04)	0.73 (0.05)

*Note*. The bold values denote the best metrics obtained by the classifiers

Table 5 illustrates the best *macro-averaged F*_*1*_* scores* with the SDs of the multi-label and single-label classifiers by every category of cognitive presence. The multi-label classifiers reached better *F*_*1*_* scores* in most of the categories. In Exploration, all the classifiers performed the highest *F*_*1*_* scores*, in which the multi-label classifiers were almost the same as the baselines (i.e., the differences $\le 0.01$). In Triggering event, the same *F*_*1*_* scores* were obtained in the multi-label classifier with the DA and the baseline classifier without the SMOTE as the second-best score. Noticeably, the multi-label classifiers with the DA method largely outperformed other classifiers in the categories with the minority instances (i.e., the Other and Resolution); however, the *F*_*1*_* score* decreased in Integration after using the DA method in the multi-label classifier.

**Table 5:** The macro-averaged F_1_ scores (the best case and SDs on the testing AgreementSet) by the cognitive presence phases

Classifiers	Other	Trigger	Exploration	Integration	Resolution
Multi-label BERT without DA	0.00 (0.00)	0.65 (0.02)	**0.85** (0.03)	**0.60** (0.02)	0.00 (0.00)
Multi-label BERT with DA	0.40 (0.05)	**0.68** (0.03)	**0.85** (0.02)	0.39 (0.03)	**0.57** (0.14)
Single-label RF without SMOTE	0.29 (0.06)	**0.68** (0.06)	**0.86** (0.06)	0.53 (0.04)	0.00 (0.00)
Single-label RF with SMOTE	0.32 (0.06)	0.61 (0.05)	0.84 (0.04)	0.51 (0.05)	0.00 (0.00)

*Note*. The bold values denote the highest F_1_ scores obtained by the classifiers

### Training on the AgreementSet and Testing on the DisagreementSet

We tested the best-performing classifiers trained by the AgreementSet on the DisagreementSet in the study. Table 6 displays the *example-based* metrics of the multi-label classifiers with and without the DA method. The *exact match ratio* and the *F*_*1*_* score* were improved by using the DA method. Compared to the results of the AgreementSet in Table 4, we found the *F*_*1*_* score* reached the similar value in the test with the DA method, but the *exact match ratio* and the *fully incorrect ratio* largely declined. Interestingly, the messages that were labelled partially correct accounted for beyond half of the entire DisagreementSet. This outcome was opposite to the results in the testing AgreementSet (Table 4).

**Table 6:** The example-based performance of the classifiers (testing on the DisagreementSet)

Classifiers	Exact match ratio %	Partially correct ratio %	Fully incorrect ratio %	Example-based F_1_ score
Multi-label BERT without DA	20.10	**76.00**	3.90	0.71
Multi-label BERT with DA	**36.30**	61.60	**2.10**	**0.77**

*Note*. The bold values denote the better metrics obtained by the classifiers

Table 7 displays the *macro-average F*_*1*_* scores* by each cognitive phase in the DisagreementSet with and without the DA method. Using DA method improved the *F*_*1*_* scores* in the Other, Exploration, and Integration phase. The multi-label classifiers reached very similar performance in the Triggering event phase. Compared to the outcomes of the AgreementSet in Table 5, the classifiers obtained overall higher *F*_*1*_* scores* in the DisagreementSet except the Resolution messages. Similarly, the classifiers performed the best in the Exploration phase, followed by the Triggering event. Also, the *F*_*1*_* scores* were near to zero regardless of using the DA method.

**Table 7:** The macro-averaged F_1_ scores (testing on the DisagreementSet) by the cognitive presence phases

Classifiers	Other	Trigger	Exploration	Integration	Resolution
Multi-label BERT without DA	0.28	**0.77**	0.88	0.59	0.03
Multi-label BERT with DA	**0.57**	**0.76**	**0.90**	**0.73**	0.00

*Note*. The bold values denote the better F_1_ scores obtained by the classifiers

**Fig. 6:**
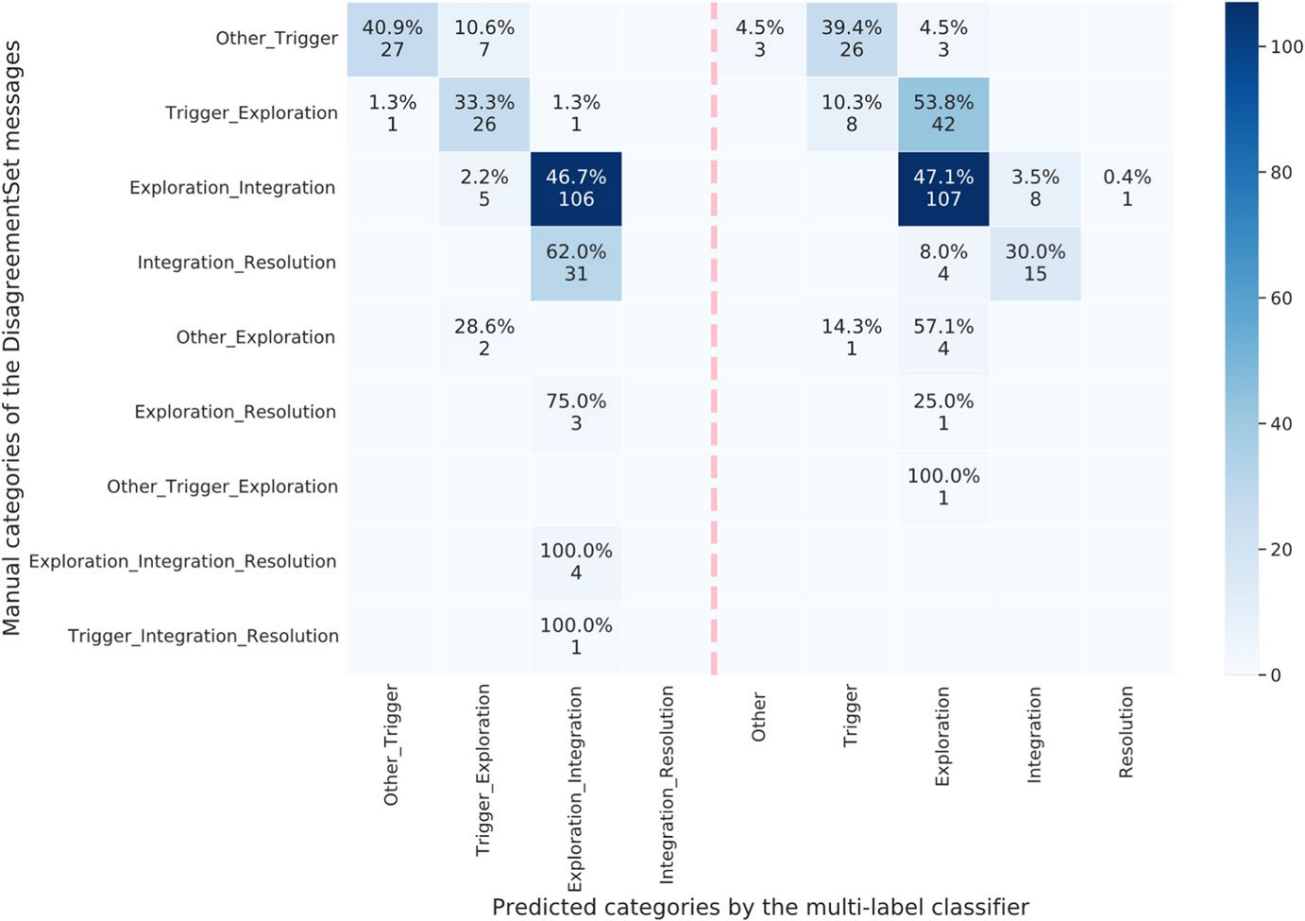
The confusion matrix using a heat map when we tested the best-performing multi-label classifiers on the DisagreementSet. The darker the blue cells, the larger the number of the predicted messages

Figure 6 depicts the confusion matrix of the prediction tests on the DisagreementSet using a heat map. The columns denote all the outcomes of the predicted labels including the combinations of multiple labels (the left of the dashed vertical line) and the one unique label (the right of the line). The rows represent the manual labels of the DisagreementSet messages, which aligned with the distribution in Table 3. The blue cells display the number of the messages with manual labels in the target row that were labelled as the target phases in the columns, together with their percentage in the rows. The darker the blue cells, the larger the number of the messages. The prediction results suggest that the multi-label classifiers labelled the DisagreementSet messages into either one unique phase of cognitive presence or two adjacent phases.

According to the above results, we answer the *first sub-question* that in the *example-based* measures, the multi-label deep learning classifiers outperformed the single-label baselines with the slightly higher *example-based F*_*1*_* scores* but under-performed with lower *exact match ratios*. Also, the multi-label classifiers outperformed the single-label baselines with the higher *macro-averaged F*_*1*_* scores* by most of the cognitive presence categories. These results may be because the multi-label classifiers allow messages to be tagged with more than one label, increasing the chance of correctness in each category. In this regard, the *exact match ratio* would decrease, and the *partially correct ratio* would grow if the boundaries between the categories were blurry. We also found that the maximum number of predicted labels was not more than two in the multi-label classification experiments including the tests on the AgreementSet and DisagreementSet, and all the two-label predictions were adjacent phases of cognitive presence. In contrast, almost 4% of the messages in the categorisation results by the expert coders (Table 3) were labelled into two non-adjacent phases or even three phases, which could be subjectively incorrect categorisation. We assume the multi-label classifiers have the potential to identify and reduce the subjectivity in manual categorisation.

Some relevance between the classification decisions of expert coders and the multi-label classifiers was reflected in the outcomes of the tests on the DisagreementSet. Compared to the results of the tests on the AgreementSet (Table 4), the *partially correct ratios* of the DisagreementSet messages (Table 6) largely increased and the *exact match ratios* decreased. The multi-label classifiers predicted more than half of the testing AgreementSet messages as one unique phase, which was the same with the decisions of the expert coders. In contrast, they labelled most of the DisagreementSet messages into two adjacent phases, amongst which one of the predicted labels was the same with the manual labels. We think that the multi-label classifiers can support the categorisation work of the expert coders. The classifiers found the exists of confusion between adjacent phases in the online discussions as the human coders have found, and may also help correct the coders’ categorisation decisions when they have very large distinctions (e.g., non-adjacent phases of cognitive presence).

The results also suggest that the DA methods have the potential to improve the class imbalance problem. The DA methods had a better effect of identifying the messages in the Resolution category than the SMOTE method. However, we found that many messages with a manual label of Exploration were tagged as both Exploration and Integration labels after applying the DA method, which could affect the *F*_*1*_* scores*. Using the DA method might increase the risk of deepening the confusion (i.e., blur area) between Exploration and Integration since most of the disagreements between the expert coders appeared between these two categories (see Table 3 and Fig. 3). We still need more instances of the very skewed category (e.g., Resolution) to improve the automatic classification performance.

### Partially Correct Predictions of Cognitive Presence on the AgreementSet by the Multi-Label Classifiers—SQ2

In this study, the *partially correct* prediction means that one of the two predicted labels was the same as the manual label, but the other was not. The *partially correct ratio* was 39.86% (Table 4) when we used the multi-label classifier with the DA method. To answer the *second sub-question*, we investigated the 39.86% messages since the experiment achieved the best performance amongst the other classifiers.

**Fig. 7:**
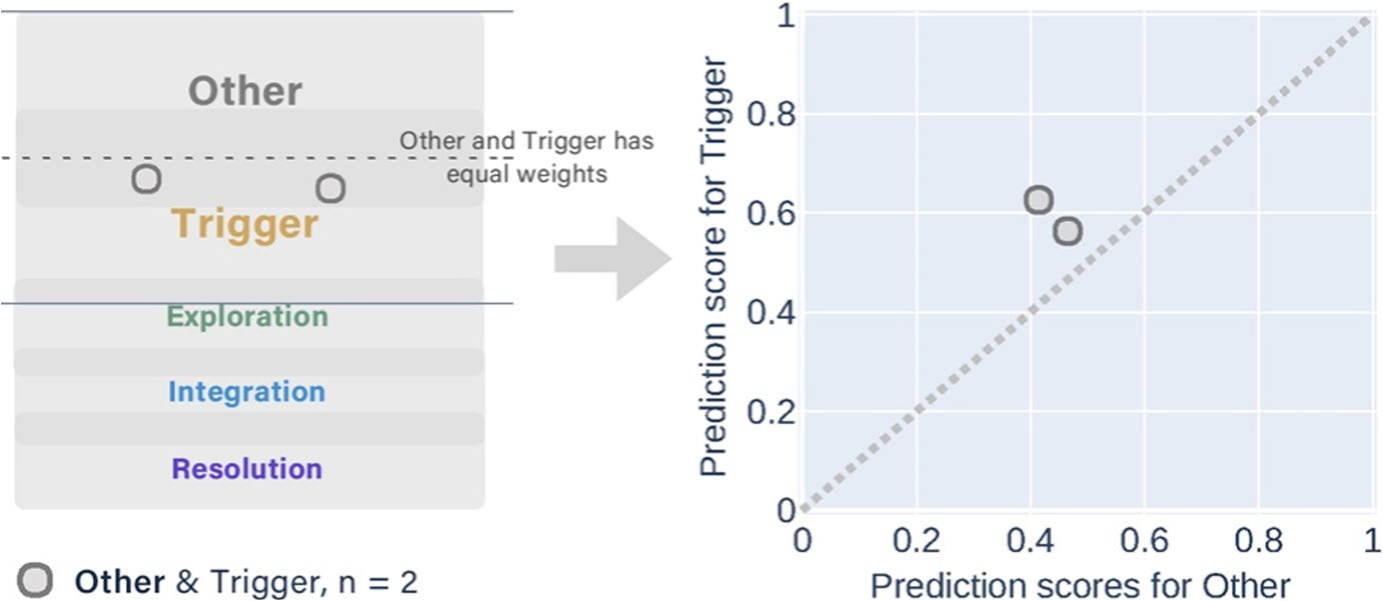
The conceptual diagram on the left simulates the predicted labels of two messages located in the overlapping area between the Other & Triggering event but their manual labels were the Other. The X–Y graph on the right shows that the probability scores of Triggering events were higher than Other with the multi-label classifier

**Fig. 8:**
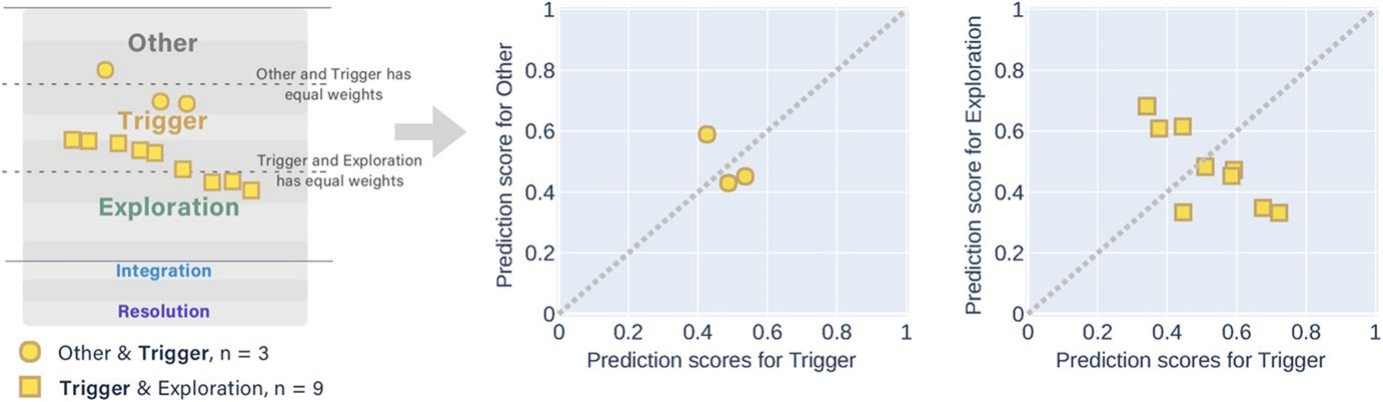
Twelve messages with the manual label of Triggering event were predicted as Other & Triggering event (3 messages), and Triggering event & Exploration (9 messages) separately

**Fig. 9:**
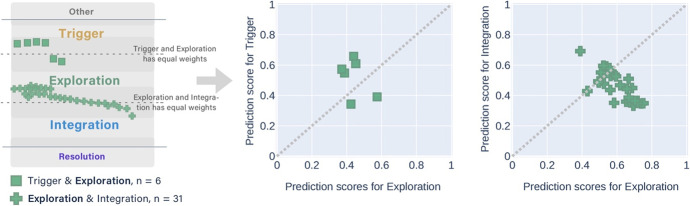
Thirty-seven messages with the manual label of Exploration were predicted as Triggering event & Exploration (6 messages), and Exploration & Integration (31 messages) separately. When the alternative label was Integration (the second X–Y graph), the messages obtained higher prediction scores in Exploration than Integration

**Fig. 10:**
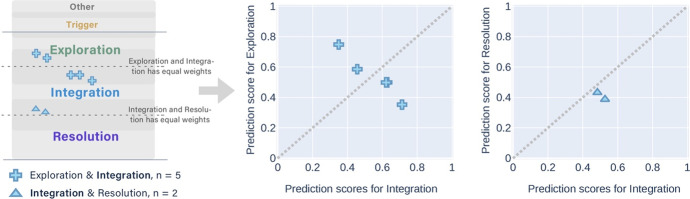
Seven messages with the manual label of Integration were predicted as Exploration & Integration (5 messages), and Integration & Resolution (2 messages) separately

**Fig. 11:**
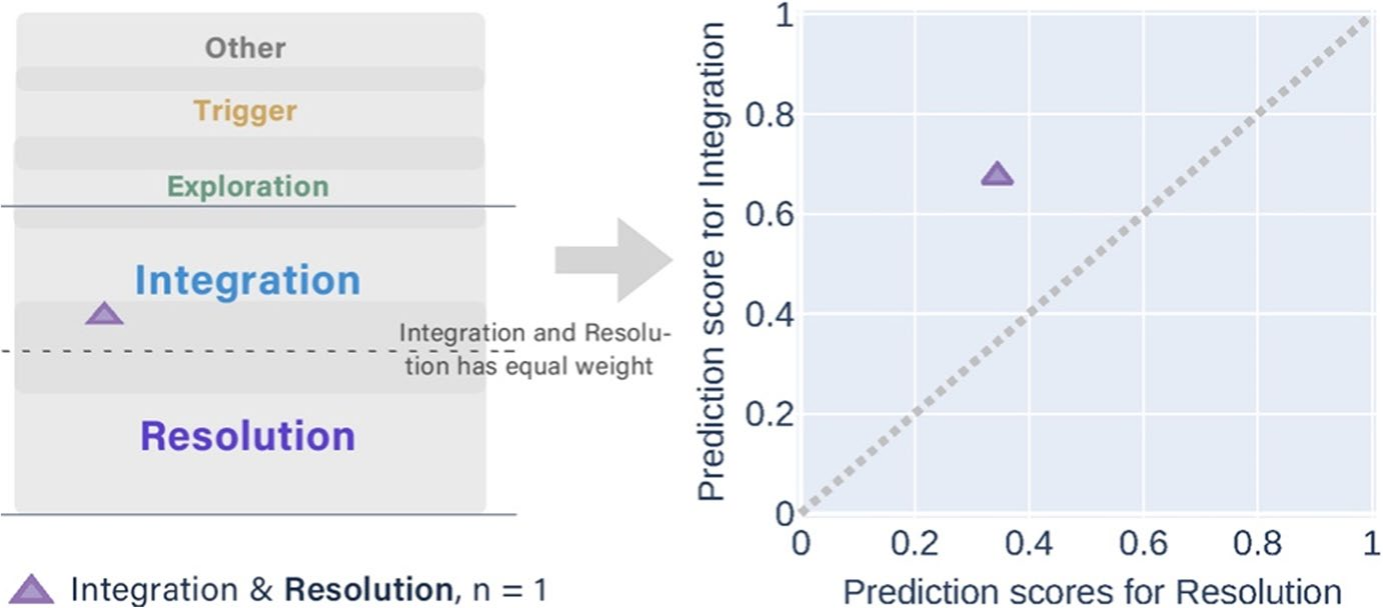
Only a message with the manual label of Resolution was predicted as a higher score in Integration and a lower score in Resolution

The *partially correct* predictions consisted of eight groups that had different combinations of manual and predicted labels. The messages that had the same manual label were displayed in one figure (Figs. 7, 8, 9, 10, and 11). In each figure, two types of scatter plots were used to demonstrate the manual and predicted labels of the messages, and a marker represents a message. The left plot simulates that the predicted labels of the messages located in the overlapping areas between the adjacent categories of cognitive presence, which is similar to the conceptual diagram in Fig. 3. The right X–Y graph demonstrates the exact probability scores (ranged from 0.33 to 1) of the two adjacent predicted labels. Five-coloured markers denote the different manual labels of the messages. The same shape of the markers was used in the plots when the combinations of the two predicted labels were the same. The dotted lines are boundaries that separate the upper and lower regions. The two predicted labels obtained the equivalent scores if the messages were exactly on the dotted line. In the X–Y graphs, the messages in the lower region were predicted to have higher scores in the x-axis categories, which were the same as the manual labelling.

Most of the *partially correct* predictions had the manual label of Exploration (Fig. 9). They had more of the alternatively predicted label of Integration than Triggering event.

The messages with the manual label of Triggering event had more alternative labels of Exploration than Other (Fig. 8). The messages with the manual label of Integration obtained more alternative labels of Exploration than Resolution (Fig. 10). Both messages that had the alternative label of Resolution achieved the higher probability scores in Integration than Exploration.

We also analysed the *partially correct* predictions qualitatively by the groups that had different combinations of manual and predicted labels. Due to the page limitation, we can only offer some typical representations in Table 7 to analyse why the multi-label classifiers predicted such labels of cognitive presence categories, particularly the higher ones.

### Predicted Labels of Both Triggering Event and Exploration

The first message in Table 8 delivered an agreement statement with a short sentence after it, which should be a “simple agreement” in Triggering event according to the adapted coding rubric (Hu et al., 2020). The classifier scored it a 0.61 probability in the Exploration apart from a 0.44 in Triggering event. This prediction might be because the second sentence can be “insufficient support for the agreement” (Hu et al., 2020), which should be in the Exploration. The second message started with a question and then intended to provide an outside resource in the thread, which should be an “information exchange” in the Exploration. The classifier also scored a 0.66 probability in the Exploration might be because the author planned to provide the external information but not yet, so it can still fit in the scope of the Triggering event. The third message stated a disagreement with a personal opinion, which should be in the Exploration. However, the second sentence also delivered a `feeling of difficulty, which was a key indicator of Triggering event. This indicator might be a possible reason that our classifier scored a 0.61 probability in Triggering event.

**Table 8:** Sample messages of the partially correct predictions by the multi-label classifier. Each message contained the texts, the manual label, and two predicted labels with the probability scores representing each group

	Message text	Manual label	Predicted label 1 (score)	Predicted label 2 (score)
1	“I totally agree with you. Morality is personally (individually)-driven”	Trigger	Trigger (0.44)	Exploration (**0.61**)
2	“Did anyone else see the exchange between a law professor and a student about a ‘Black lives matter’ t-shirt? The professor wrote a brilliant response that is relevant to this course. I will try and paste it below.”	Exploration	Trigger (**0.66**)	Exploration (0.44)
3	“Yes you could right, but I constructed this from the letter writer argument lr point of view. I found the exersise difficult.”	Exploration	Trigger (**0.61**)	Exploration (0.45)
4	“I understand the distinction to be this: deductive reasoning is reasoning from generally accepted principles or known facts, whereas non-deductive reasoning is reasoning from specific instances or past experiences. Example of deductive reasoning: P1: The constitution guarantees all citizens the right to vote P2: Women are citizens Therefore, C: The constitution guarantees women the right to vote Example of non-deductive reasoning: P1: My Critical Thinking course was difficult P2: My best friend’s Critical Thinking course was difficult, Therefore, probably, C: Critical Thinking courses are difficult”	Exploration	Exploration (0.56)	Integration (0.53)
5	“P1: Animals from factory farming are treated cruelly. P2: Eggs come from hens that are treated cruelly…Therefore, C: You should go vegan Justin makes a deductive argument on why you should go vegan but it is invalid. While the premises aren’t false, the problem is that they do not support the conclusion that a person should go vegan. Rather, they would support a conclusion as to why you should not buy products made from factory farms, not adopt a new dietary philosophy. A person could get their meat and animal products from farms that ethically raise their animals.”	Integration	Exploration (0.50)	Integration (**0.62**)
6	“You make the point that the authority to which an appeal is made, must be competent and have sufficient expertise. I have thought about this but surely if they, whoever they are and whoever they represent, are represented to us as the authority, we have to assume that they are also the experts and that there are no resources above them—that is what the authority are there for—‘aye but there is the rub’ as Shakespeare said. There is no discernible proof that they are expert, that in fact we may be in a better position than they, factually. I may have to go to the ‘Tax’ Office as my authority in the matters of my taxes but who is to say that they are any more knowledgeable that we are. However we are forced to so because they are the ‘authority’.”	Integration	Integration (**0.53**)	Resolution (0.39)
7	“I don’t agree. Of distinction is not whether you have to take the exact circumstances into account. It is how large the range of permissable actions are in that exact situation. To take a concrete example. The exact situation is you walking down the main Street of your home city tomorrow and passing a begger. Your options contain; A) Give the begger some money. B) Ignore the begger… An moral realist might say that it is morally required to do option A. A pluralist might say either option A or B is acceptable. A complete relativist will say no options are morally better than any others. These are not exact terms though and and a particular variety of moral realist might argue that there are degrees of rightness or a range or morally right options that are coherent with the one correct set of values. It also might make sense to argue that most actions do not have a moral component so relativism is mostly true."	Resolution	Integration (**0.67**)	Resolution (0.34)

*Note*. The bold values denote the higher prediction scores for each message

### Predicted Labels of Both Exploration and Integration

The fourth message in Table 8 made a personal clarification about two course related concepts and provided examples, which should be an “information exchange” in the Exploration. Our classifier also scored a similar 0.53 probability in Integration might be because it contained the typical language structures of conclusions with supporting ideas (e.g., “Premise1…Premise2…(Probably) Therefore…”). The fifth message used the same structure to explain an example as the fourth message, but it provided further elaborations to support the main conclusion and solution in the last two sentences. According to the adapted rubric (Hu et al., 2020), it was manually labelled into Integration. However, the multi-label classifier also scored it a 0.50 probability in the Exploration. We assume that the classifier algorithm might ‘think’ these two messages had similar language structures.

### Predicted Labels of Both Integration and Resolution Phases

The sixth message constructed a new understanding of a concept in the course (e.g., authority) by building on the conclusion of other group members, which should be a “convergence with supporting ideas” in Integration. The classifier also predicted a slight probability of 0.39 in Resolution. The seventh message proposed a divergent opinion from another learner’s conclusion with a concrete example. Also, it offered deeper thoughts based on the previous conclusion, which should be in Resolution. The classifier tagged it a low probability of 0.34 in Resolution but a higher probability of 0.67 in Integration. The classifier tends to regard these two messages as similar patterns. Objectively, these two messages might not have clear boundaries if we rethink the definition of ‘new’ or deeper constructions as the paramount indicators of Resolution in the Garrison et al. (2001) and Hu et al. (2020) coding rubrics of cognitive presence. The NLP and deep learning algorithm might consider them as a ‘wider conclusion’.

To summarise, we answer the *second sub-question (SQ2)* that the best-performing multi-label classifier tagged almost 40% messages of the testing set into two adjacent labels in the cognitive presence categories, in which one of them was the same as their manual labels. We also found that the messages with the manual label of the start (the Other) or the end (Resolution) of a cognitive presence cycle had only one alternatively predicted label other than the manual label. The message with the manual label that occurred at the middle of the cycle (i.e., Triggering event, Exploration, and Integration) had two alternatively predicted labels, either before or after the manual one. This distribution was similar to the disagreements between the three coders (Table 3 and Fig. 3). Notably, the multi-label classifier did not tag any messages into non-adjacent labels. In contrast, the three coders classified a small fraction of the messages into non-adjacent or three labels (Table 3). The non-adjacent labels can be triggered by the coders' subjectivity as the ‘outliers’. The prediction results imply that the multi-label classifier could identify the subjectivity in the manual categorisation.

Most of the two-label predictions appeared between Exploration and Integration, which was also aligned with the coders’ disagreements (Table 3 and Fig. 3). From the qualitative analysis, we found that these messages often contained the condition-and-conclusion structures that express learners’ opinions using sentences such as “*Premise1… Premise2… (Probably) Therefore…*”. The structure can be closely associated with the definitions and indicators of Exploration and Integration in the coding rubric (Hu et al., 2020). During both the cognitive phases, learners proposed their solutions or conclusions. In the former phase, the conclusion was not coherent, whilst in the latter, it was coherent with sufficient support. The coders differentiated the two categories through their subjective understanding of whether the logical reasoning was sound. However, the NLP and deep learning algorithms identify the categories through analysing the language patterns as indications. In other words, the algorithms label a message according to the computational probability that similar language patterns of a label appear in the message. From the qualitative analysis of the sample messages, we assume that the multi-label classifiers granted a probability score of the cognitive presence category to a message when the indicators (i.e., common language patterns) of the category appeared to some extent. Hence, we think the “multiplicity” way of the multi-label identification could be less subjective than the “dualist” way (e.g., the conclusion is coherent or not) of the manual categorisation (Perry, 1999).

### Partially Correct Predictions of Cognitive Presence on the DisagreementSet by the Multi-Label Classifiers – SQ3

We also investigated the messages of the partially correct predictions when we tested the best-performing classifiers on the DisagreementSet, especially the messages that had adjacent manual labels but were predicted as one unique category. We wonder if any patterns can be found in these messages. We draw a comparison between these messages and the messages that were predicted as exact correct in the DisagreementSet. Table 9 tabulates the mean and SD values of some textual and contextual features extracted in the messages. The textual features contain the number of words, the number of sentences, and the measure of textual lexical diversity (MTLD). The MTLD was calculated as the mean length of word strings that maintained a criterion level of lexical variation in each message (McCarthy & Jarvis, 2010). The contextual features include the depth (conversation level) of a message in its thread, and the number of replies after the current message. We exclude the messages that had less than ten predictions in the DisagreementSet tests (details in Fig. 6) as the numbers were too few for the statistical calculation. We then discussed the statistical results (Table 9) and analysed the example messages qualitatively in the order of their manual labels.

**Table 9:** Summary of the textual and contextual features of the DisagreementSet messages that contained two adjacent manual categories but were predicted as partially correct (i.e., only one of the labels was the same)

Manual categories	Predicted categories	Number of words	Number of sentences	MTLD*	Depth	Replies	Messages n
		Mean (SD)					
Other & Trigger	Other & Trigger	7.56 (5.45)	1.30 (0.47)	2.29 (8.30)	3.15 (2.61)	2.33 (6.13)	26
		e.g., *“I really like your comments.”*					
	Trigger	13.65 (9.09)	1.62 (0.85)	13.12 (27.83)	5.88 (6.19)	3.04 (7.12)	27
		e.g., *“I really like the gender-sensitivity of the article in the last paragraph.”*					
Trigger & Exploration	Trigger & Exploration	29.08 (15.31)	2.23 (1.11)	62.14 (44.29)	3.88 (2.92)	0.96 (1.11)	26
		e.g., *“I personally agree with his premises. And I am vegetarian. For 20 years.”*					
	Exploration	40.95 (21.24)	2.19 (1.21)	64.13 (41.73)	3.93 (5.29)	1.83 (2.77)	42
		e.g., *“I do not disagree, and I am fully aware that I cannot make people think as I do (even if I assume my own thinking is right). But it does raise and interesting or important moral dilemma.”*					
Exploration & Integration	Exploration & Integration	120.30 (51.24)	5.36 (3.45)	81.29 (28.36)	3.69 (4.26)	3.25 (4.04)	106
		e.g., *“Example – Premise 1: Mammals are warm blooded. Premise 2: Fish are not warm blooded Conclusion: Therefore fish are not Mammals. This argument is deductive, valid and sound.”*					
	Exploration	70.50 (35.72)	3.45 (1.75)	94.52 (0.08)	4.00 (3.98)	3.39 (5.26)	107
		e.g., *“Personally I think this section is only constructive if one leaves ones personal beliefs aside and addresses the key issues without reference to any specific religion.”*					
Integration & Resolution	Exploration & Integration	156.13 (51.82)	7.58 (3.74)	92.67 (32.16)	5.42 (5.16)	5.65 (6.79)	31
		e.g., “Ok, let me try to boil down your version to its simplest form: Premise 1: Old people have been wrong about new music in the past. Therefore: Their complaints about new music now are unfounded. I still see this as really problematic, because its possible their complaints are *not* unfounded, but there’s no way of knowing that until you have looked at the reasons (or lack thereof) for the complaints themselves.”					
	Integration	196.47 (38.90)	8.13 (2.89)	76.76 (19.32)	5.27 (5.74)	5.80 (5.87)	15
		e.g., *“Scientific argument—This is based off of the information given above and without trying to over complicate or think things too much. Even though there was reform in schools from the 70's onward with regards to how left handed children were treated or seen, this is unlikely to have had any effect on many of the presidents or candidates. This leaves the notion that left handed people have a more determined nature and perhaps other characteristics which have lead them to politics. Premise 1. S—More than half of the past 14 presidents are left handed which is above average for the US; why is this? Premise 2. H—People who are left handed develop particular personality traits which lend themselves to a career in politics. Premise 3. No other Hypothesis can explain S, given the information above, better than H Therefore, probably therefore. H is true. To develop this further a set of tests would have to be carried out in order to conclude that the hypothesis is either correct or in correct.”*					

*MTLD denotes the measure of the textual lexical diversity (McCarthy & Jarvis, 2010). The values contain the means and SDs

### Manual Categories of Both the Other and Triggering Event

The multi-label classifiers predicted 40.9% of the messages that had the manual labels of both the Other and Triggering event (the first row in Fig. 6) as the same two labels. Another close percentage (39.4%) of the messages were predicted partially correct, as the Triggering event only. We read the messages in the two groups and found two common patterns: 1) most of them consisted of one or two sentences, which can also be revealed in the first two rows in Table 9; 2) many of them contained compliment expressions to the course or other users’ comments, such as “I enjoyed this course”. We also found two distinctions: 1) the numbers of words and the lexical diversity used in the former group were averagely fewer than those in the latter group (Table 9), which means the messages predicted only one category, the triggering event, used more complex words than the messages predicted two categories; 2) the compliment sentence in the majority of the messages from the former group was delivered in a general way, such as “I really like your comments”, whereas in the latter group the compliment often contained a specific subject, such as “I really like the gender-sensitivity of the article in the last paragraph”.

### Manual Categories of Both the Triggering Event and Exploration

We found that 33.3% of the messages that contained the manual labels of both Triggering event and Exploration (the second row in Fig. 6) was predicted as the same two labels. A percentage of 53.8% messages was predicted into the single Exploration category. In Table 9, the average number of sentences and lexical diversity measures shown were similar between the two groups, but the messages in the former group used fewer words and had smaller differences in the message depth and number of replies than in the latter group. We also found that most of the messages in the two groups contained (dis)agreement statements on other users’ messages by reading the messages carefully. Interestingly, the messages in the former group often used concise messages or incomplete sentences as the supports behind the (dis)agreement opinions. Conversely, the messages in the latter group often contained longer and complete supporting ideas.

### Manual Categories of Both the Exploration and Integration

The group of messages that contained the two predicted labels (Exploration and Integration) accounted for 62% in the messages that had the same two manual labels in the third row in Fig. 6. Another group of messages that were predicted as one label, Exploration, reached a similar ratio of 47.1%. We found the former group of messages used the words of averagely larger numbers but lower lexical diversity than the latter one in Table 9. After reviewing these messages, we found that a common structure to claim users’ opinions frequently appeared at the messages in the former group; that was, “*Premise 1…Premise 2…(Probably) Therefore…*”. However, this expression seldom occurred in the messages from the latter group.

### Manual Categories of Both the Integration and Resolution

The multi-label classifiers predicted 62% of the messages that had the manual label of both Integration and Resolution (the fourth row in Fig. 6) as other two labels, Exploration and Integration, and another 30% of the messages as the single Integration category. No messages were predicted as the exactly correct two labels. The messages in these two groups obtained very close values of the number of sentences, the message depth, and the number of the replies, which can be found in Table 9. The former group of messages contained fewer tokens and sentences, but higher lexical diversity values than those in the latter group. We also found many messages in the two group had the premise-and-conclusion structure as the two-label prediction messages in the previous group (the third row in Fig. 6).

According to the above results, we answer the *third sub-question (SQ3)* that the best-performing multi-label classifier predicted 51.1% of the DisagreementSet messages as one single category of cognitive presence, and 96% of these single-label predictions was the same as one of the expert coders’ decisions. Meanwhile, all the predictions of the DisagreementSet messages were either one single phase or two adjacent phases of cognitive presence, even the messages that were categorised to two non-adjacent phases or three phases (the last three rows in Table 3). The prediction results of the DisagreementSet were consistent with the tests in the AgreementSet. Also, the multi-label classifiers predicted almost half of the DisagreementSet messages as two labels of Exploration and Integration, and approximately another half as the single Exploration category. This distribution was aligned with the expert coders’ main disagreements, amongst which the highest proportion also fell between Exploration and Integration categories, the middle processes of cognitive presence. These results suggest that the multi-label classifiers encountered the same challenge as the expert coders had faced to differ the Exploration from Integration messages in MOOCs (Hu et al., 2021a). It might because that these confused messages applied very similar linguistic and contextual features. We found that the premise-and-conclusion expressions in the messages could be a strong indicator for the multi-label classifiers to identify Integration category. Also, the number and the lexical diversity of the words used in the messages could be strong indicators for the multi-label classifiers to make a decision between the lower phases; for example, to identify Triggering event from the Other phase, and Exploration from Triggering event. This finding was in line with the previous studies using the single-label classification models (Barbosa et al., 2020; Farrow et al., 2020; Kovanović et al., 2016; Neto et al., 2018). Nevertheless, the two features tended to be very weak indicators to identify the adjacent higher phases, such as Integration and Resolution.

## Summary of Discussion

All the above results suggest that although learners’ cognitive presence develop from shallow to deep phases, the defined boundaries between the phases could be blurry in the MOOC discussion messages. If the boundaries between the adjacent categories were clear, the multi-label classifiers would not tag many messages into two labels. Some MOOC discussion messages were difficult for both human coders and computer algorithms to differentiate, especially between Exploration and Integration. Some discussion messages were clearer for both humans and computers to accurately allocate one unique category of cognitive presence. These phenomena may be due to the messy and chaotic reality of online discussions, especially in MOOCs, which consists of conversational flows that do not fit into the ordered patterns in the CoI (Xin, 2012). It is nearly impossible for the limited number of researchers to read the myriad of MOOC discussions qualitatively and objectively without computer support. In this regard, computer algorithms are required to support researchers to discover the general trend in massive data and make wiser decisions. The coders and researchers can rethink their disagreements and make less subjective decisions on the cognitive presence categorisation according to the results of multi-label classifiers. Also, the generative machine learning models that focus on the likelihood estimation of data distribution (e.g., unsupervised learning methods) can be recommendable for the cognitive presence categorisation rather than the discriminative models, which aim to seek the decision boundaries of the classes (e.g., the random forests). Finally, our findings can inform the ongoing refinement of the CoI framework to accommodate the MOOC context. Additional categories can be included between the adjacent categories of cognitive presence to accommodate the predominant disagreements between coders and the confusion of the automatic classifiers. The cognitive presence scheme could learn from Perry’s scheme of intellectual and ethical development (Finster, 1989; Perry, 1999), where the stages are developmental including several positions and tolerate the overlaps existing between adjacent stages. We suggest that in the manual categorisation process, the coders can tag multiple labels of cognitive presence phases with their confidence degrees on each discussion message rather than an absolute, unique label in the previous studies. For example, a message can be tagged both Exploration and Integration with the confidence degree of either 1 (i.e., ‘not sure’), 2 (i.e., ‘half sure’), or 3 (i.e., ‘sure’), respectively. Training the automatic classifiers can also benefit from the multiple labels with their degree values to help reduce subjectivity and tolerate the multiplicity in the manual classification tasks in future studies.

The outcomes of the experiments in the paper also indicate that the performance of the automatic classifiers might have some relevance to the distribution of the cognitive presence phases. The classifiers in the study reached the similar level of prediction performance to the classifiers in most of the previous studies in the literature (Barbosa et al., 2021; Farrow et al., 2019; Hu et al., 2021b; Kovanović et al., 2016; Waters et al., 2015). We found the cognitive presence phases in the training set (AgreementSet) of this study obtained very similar distribution to the data sets in the previous studies, where the highest proportion located in Exploration and Integration, and the lowest in the Other and Resolution. In contrast, distinct distributions of cognitive phases in Lee et al.’s (2022) work, where bulk of messages located in the Other and small fractions in Triggering event and Exploration, performed a very high performance of *F*_*1*_* scores*. We acknowledge that the different course domain and pedagogical decision could affect the distribution of learners’ cognitive presence (Lee et al., 2022). We suggest future work can apply the discussion messages from the MOOCs of different designs and domains, which contain diverse distributions of cognitive presence, to improve the automatic classifiers.

To summary, in response to the main research question (*What would the prediction performance of using the multi-label classifiers to categorise the phases of cognitive presence, and what can we learn about cognitive presence in the MOOC discussion messages from the training and test processes?*), we conclude that the multi-label classifiers slightly outperformed the state-of-the-art single-label classifiers, and they predicted the messages that had been categorised differently by the expert coders into one single category or two adjacent categories of cognitive presence. We envisage that overlaps exist between the definitions the adjacent phases of cognitive presence, and the multi-label deep learning method has the potential to identify the subjectivity of cognitive presence categorisation in the discussion messages from the target MOOC.

## Limitations

We acknowledge the limitations of the discussion data used in this study. The automatic classifiers developed for a specific MOOC might not be generalisable to other courses. There are disciplinary and pedagogical-design differences in the utterances that reflect learners' cognitive presence (Lee et al., 2022). Also, the limited size of the sample data and the unbalanced classes could affect the prediction performance of the automatic classifiers. We are aware that the research findings might only be valid for the cognitive presence categorisation in the target MOOC. A larger sample data size and more data from other domains will advance the performance of the automatic classifiers and its generalisability in more diverse contexts in our future work.

## Concluding Remarks

This study makes two contributions. First, we developed a multi-label, fine-tuning BERT model as a triangulation process to classify cognitive presence in the discussion messages from a philosophy MOOC. The classifier achieved slightly better performance in the *F*_*1*_* scores* than the state-of-the-art, single-label classifier. Second, we analysed the partially correct predictions quantitatively and qualitatively in both the AgreementSet and DisagreementSet to interpret how the multi-label classifiers made the decisions. Although the improvement of the classification performance is marginal, the main contributions of the study inform that the blur boundaries exist between the adjacent categories of cognitive presence in the MOOC discussion messages, and the multi-label classifiers have the potential to help researchers identify research subjectivity and make better descions in the manual categorisation of cognitive presence.

We recommend that to improve the prediction performance of the multi-label classifiers, and to inform the ongoing refinement of the CoI framework in MOOCs, future research could 1) address the class imbalance problem before the model training process by investigating effective methods (e.g., active machine learning (Rubens et al., 2015)) to automatically and adaptively generate pre-labelled data, which can reduce the time and labour of the manual categorisation; 2) adapt the categorisation instruments of cognitive presence that enable coders to tag the messages into multiple labels with confidence degrees as a significant foundation for developing automatic classifiers, which can support educators and researchers to get a richer and better understanding of cognitive presence in MOOC discussions. They can also be applied to support educators in monitoring learners’ progress and help learners self-assess in real-time, as well as implementing these tools on learning platforms at a large scale.

## Footnotes

1 https://www.futurelearn.com/courses/logical-and-critical-thinking

## Declarations

The owner of the Philosophy MOOC data used in the study are the enrolled learners who posted and commented in the LCT MOOC on the FutureLearn platform. The users granted their permission for using the data for research purposes during the course enrolment process according to the research ethics policy on the platform. The research proposal received ethical approval from the the University Human Participants Ethics Committee, and the original study data are not open.
